# Prolonged treadmill training is not able to prevent ovariectomy-induced bone loss

**DOI:** 10.3389/fphys.2022.1078857

**Published:** 2022-12-16

**Authors:** Tim Massing, Konstantin Will, Michael Müller, Johann Aleith, Tobias Lindner, Mareike Warkentin, Brigitte Müller-Hilke, Thomas Mittlmeier

**Affiliations:** ^1^ Department for Trauma, Hand and Reconstructive Surgery, Rostock University Medical Center, Rostock, Germany; ^2^ Core Facility for Cell Sorting and Cell Analysis, Rostock University Medical Center, Rostock, Germany; ^3^ Core Facility Multimodal Small Animal Imaging, Rostock University Medical Center, Rostock, Germany; ^4^ Department of Material Science and Medical Engineering, Rostock University, Rostock, Germany

**Keywords:** treadmill, ovariectomy, osteoporosis, physical training, mouse, micro-computed tomography, bone turnover markers

## Abstract

**Introduction:** Exercise is widely recognized as prophylaxis for osteoporosis. However, exactly which type of exercise is best to prevent loss of bone mass remains undefined. To find an appropriate form of treadmill exercise that would ameliorate postmenopausal loss of cortical and trabecular structures, we compared various training regimen in ovariectomized (OVX) C57BL/6J mice.

**Methods:** Common to all regimen were training durations of 14 weeks including five 30 min-sessions per week. Two groups—one sham operated, one OVX—served as controls that did not perform any training. Three OVX groups ran at constant speed, either without any incline or at 20° in- and 20° decline, respectively. An additional OVX group ran an interval training, an alternation between intensive tempo sections and so-called slower regeneration phases. Femoral and humeral bone structures were assessed *via* micro-computed tomography (μCT), biomechanical stability of the femora *via* 3-point bending test, muscle volumes of the posterior extremities *via* magnetic resonance imaging (MRI), and bone metabolic parameters *via* ELISA on peripheral blood.

**Result:** OVX resulted in loss of bone mass and stability and a transient rise in the N-terminal collagen type I pro-peptide (PINP). Training resulted in increased muscle volumes of the heart and the lower extremities as well as increased running velocities. However, none of the exercise programs was able to prevent ovariectomyinduced loss of bone mass.

**Discussion:** These data therefore suggest that axial loading and tensile strain do not suffice as prophylaxis for postmenopausal osteoporosis yet may need to be complemented by low dose pharmaceutics or dietary supplements.

## 1 Introduction

Osteoporosis is the most common bone disease in humans, and it is estimated that worldwide, one in three women and one in five men over the age of 50 will experience an osteoporotic fracture. Postmenopausal osteoporosis is a form of primary osteoporosis. The exact aetiology is unknown. However, the decline in estrogen levels after menopause appears to be one of the possible causative factors (Hernlund et al., 2013). It is defined as “a [silent] skeletal disease characterized by reduced bone strength leading to an increased risk of fracture. Bone strength reflects the integration of two main characteristics: bone density and bone quality” (NIH Consensus Development Panel on Osteoporosis Prevention, Diagnosis, and Therapy, 2001). These fractures can have serious impacts on quality of life and even death. In the context of demographic change, a further significant increase in the number of osteoporosis cases and associated fractures is expected (Camacho et al., 2020). Pharmacological therapy of osteoporosis largely relies on bisphosphonates and these have been associated with serious side effects (Khan 2008). Measures to reduce or even replace pharmacological measures for prevention of osteoporosis are therefore gaining attention.

Leading international guidelines define immobility as a risk factor for the development of osteoporosis and therefore recommend exercise as a preventive measure. Along these lines, an Australian study showed that high-intensity resistance and impact training (HiRIT) had a significant positive effect on bone structure in postmenopausal women (Watson et al., 2018). However, the exact type and amount of physical activity that will prevent deterioration of the bone are yet ill defined (Hernlund et al., 2013; Kanis et al., 2019; Camacho et al., 2020). Based on positive experience of our research group in using mice for the model of postmenopausal osteoporosis, we deliberately decided to use C57BL/6J mice. However, the literature on exercise studies in mice is still inconclusive. For example, four and 8 weeks of sprint interval training (SIT) in young and healthy female C57BL/6J mice did not result in a significant difference between untrained and trained mice—neither for cortical nor trabecular bone masses. In male C57BL/6J experimental animals, SIT even resulted in a significant loss of cortical bone mass, consistent with a site-specific reduction of cortical bone mass (Hollinski et al., 2018). Similarly, a total of 8 weeks of high-intensity interval training with different gradient angles (-10/0/10°) had no effect on cortical and trabecular bone parameters of female STR/ort mice (Koenen et al., 2017). Furthermore, low-to-moderate intensity interval training for 5 weeks failed to prevent significant cortical and trabecular bone loss in ovariectomized C57BL/6J mice (Latza et al., 2020). In contrast, 4 weeks of endurance training in female Hsd:ICR mice led to significant improvements in cortical bone structures of femur and tibia (Wallace et al., 2015). Also, significant improvements in bone mass and quality were observed in male C57/BL6 mice after 5 weeks of endurance training with an incline of 5° (Zhang et al., 2020). Overall, the scientific literature shows very contradictory results, especially when considering the model of postmenopausal osteoporosis. Furthermore there is simply a lack of scientific data on a significantly longer training period and its effects on bone structure. In addition, we here introduced 20° inclinations and declinations, in order to investigate the impact of tensile strain and axial load, respectively. To do justice to the consideration of postmenopausal osteoporosis as a systemic disease, in addition to the pure morphology of the bone, its stability and metabolism were also methodically investigated.

## 2 Materials and methods

### 2.1 Study design

**FIGURE 1:**
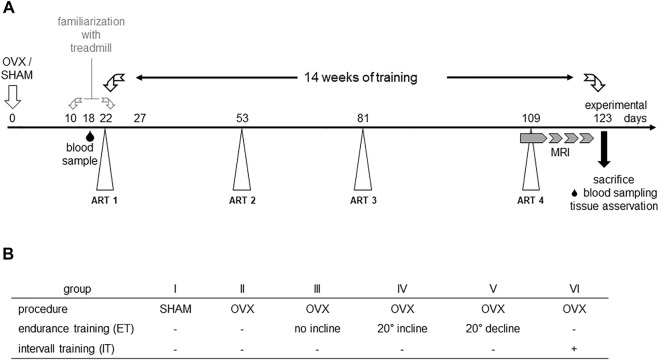
Experimental scheme **(A)** and study groups **(B).** OVX: ovariectomy, sham: sham operation, ART: Augmentation Run Test, MRI: magnetic resonance imaging.

A total of 42 7–9 weeks old female C57BL/6J mice were obtained from Charles River (Charles River Laboratories, Sulzfeld, Germany). Initially, the experimental animals had a week to acclimate to the new environment. Animals were housed in groups of six animals per cage under a 12h/12 h day and night cycle, were given water and food *ad libitum* and materials for enrichment. Randomization of animals to the six experimental groups was performed using a random number generator (Microsoft Excel 2010; Microsoft, Redmond, WA, United States). Group 1 underwent SHAM surgery and the remaining groups II-VI were ovariectomized (Figure 1). After that, the animals had 1 week to recover from the surgery. At the end of the postoperative recovery phase, groups II-IV were familiarized with the treadmill for 2 weeks. Before starting the treadmill training on experimental day 18, blood samples of the animals were collected by puncture of the great saphenous vein. Mice from groups II-VI performed a first augmentation-run-test (ART) to determine the maximum speed (Vmax), individual animals were capable of running. During the following 14 weeks of treadmill training, three more ARTs (day 53, 81, and 109) were performed to monitor changes in Vmax (Høydal et al., 2007) and to adapt the interval training accordingly (group VI). In the last 3 weeks of training, muscle volumes of the lower extremities were measured by 7 T small animal MRI. After 14 weeks of training, the animals were euthanized by cervical dislocation, blood, organs and bones of the lower extremities were harvested and preserved for post-mortem analyses. All experiments were performed in accordance with the current guidelines and scientific findings of the Society for Laboratory Animal Science (GV-SOLAS). Permission was granted by local authorities, i.e. the Landesamt für Landwirtschaft, Lebensmittelsicherheit und Fischerei Mecklenburg-Vorpommern, Germany and was registered under 722.13-1-055/19.

### 2.2 Ovariectomy

Animals were anesthetized by intraperitoneal injection of ketamine/xylazine (100 mg ketamine and 5 mg xylazine/kg body weight) and depth of anaesthesia was ensured. Before surgery, panthenol ophthalmic ointment was applied to protect eyes from dehydration, and mice were positioned on a warm plate (37°C) to protect them from hypothermia. After abdominal positioning, the lower back was shaved in the area of the surgical field followed by skin incision and opening of the peritoneum to expose uterus, tubes, and ovaries. Removal of tubes and ovaries under microscopic view (Leica Biosystems, Dusseldorf, Germany) was performed by electrocautery, followed by suturing of peritoneum and skin (Ström et al., 2012). Metamizol was added for 5 days post-surgery to the drinking water and daily scoring was used to assess animal distress. During post-mortem organ removal, uterine weight was determined in relation to body weight to verify successful ovariectomy. One mouse had to be excluded from further analyses due to incomplete ovariectomy.

### 2.3 Augmentation Run Test

Augmentation Run Tests were performed to determine the maximum velocity of experimental groups II-VI (Hollinski et al., 2018; Latza et al., 2020) and to adapt the interval training to the measured maximum speed of the group (Høydal et al., 2007). In detail, ART started at a speed of 0.17 m/s for 3 minutes. Subsequently, the speed was slowly increased to 0.2 m/s over an interval of 2 min and held for another 3 min. Thereafter, the speed was increased to 0.25 m/s over a period of 2 min and again held constant for 3 min. Subsequently, the speed was again increased by 0.05 m/s over a period of 3 min and held for 2 min before the next cycle started. The maximum speed reached without external assistance was defined as Vmax (Ingalls et al., 1996). Over the whole experimental period, four augmentation run tests were completed to monitor the effect of the training on the animals’ maximum velocity (Figure 1).

### 2.4 Training modalities

Treadmill training was performed 5 days a week from Monday to Friday for 30 min each on a six-track treadmill (TSE Systems Inc., MO, United States). Endurance training of groups III-V was accomplished at a constant speed of 0.2 m/s, but with different inclination angles of the treadmill. Thus, group III trained without any gradient, group IV trained at 20° of incline and group V at 20° of decline. Group IV also trained without any gradient yet underwent interval training adapted to their maximum velocity. In detail, mice ran at 40% of their Vmax for the first 6 min and then for four cycles of 80% of Vmax for 3 min followed by 40% of Vmax for 1.5 min.

### 2.5 Magnetic resonance imaging

To quantify the training effects on the muscle volume, MR-images of the abdomen, pelvis and lower extremities were taken. In order to ensure high quality images, the animals were sedated using isoflurane, restrained on the MRI table, continuously warmed and breath monitored. For muscle imaging T2 weighted Turbo Rapid Acquisition with Relaxation Enhancement sequences (TurboRARE) (BioSpec 70/30, Bruker, Ettlingen, Germany) were used on a 7 T MRI with the following settings: TE/TR 25.25/3227 m, field of view: 28 μm × 21 mm, matrix size 233 × 175, slice thickness 0.85 mm, resolution 120 μm × 120 μm, and RARE factor: 8, 72 mm volume resonator. All measurements were respiration triggered. Since muscle volume is also affected by individual mouse size, it was related to femur length for statistical comparison. Based on the number of slices in μCT imaging, the length of each femur was calculated using CT Analyzer (Bruker, Billerica, MA, United States). Lower extremity muscles were manually marked slice-wise using ITK SNAP software (version 3.8.0) and muscle volume was also automatically calculated by the program (Feng et al., 2014).

### 2.6 Micro-computed tomography

*Ex vivo* μCT images of right femora and right humeri were taken to evaluate the cortical and trabecular bone structure (Bruker, Billerica, MA, United States) Software Version 4.2, 0.5 mm Al-filter, integration time of 1.5 s, isotropic voxel size of 9 μm at 49 kV and 200 μA, rotation step of 0.5°, and averaging frame of 3). Femora and humeri were fixed in 4% PFA solution, and preserved in ethanol. To prepare the specimens for μCT imaging, bones were washed three times with distilled water and stored in 0.9% sodium chloride solution at 4°C over night for rehydration. During each measurement procedure, two hydroxide apatite phantoms of known density (0.25 and 0.75 g/cm^3^) were analysed in parallel in order to calibrate for the determination of bone mineral density (BMD). The CT scans were generated in a 360° scan to allow high quality and avoid artefacts. Using NRecon software (Bruker, Billerica, MA, United States), primary processing of the micro-CT data was accomplished using a Gaussian filter with a smoothing parameter of 2, x-ray hardness correction of 30%, ring artefact reduction by a factor of six and defect pixel masking of less than 20%. Homogeneous and adequate orientation of the bones for subsequent analyses was achieved using the DataViewer (Bruker, Billerica, MA, United States) software. Once the reconstruction was complete, the actual analysis of femora and humeri followed, using CT Analyser software and appropriate algorithms. In order to define regions of interest (ROI) for femora the distal metaphyseal growth plate and the fusion zone of the greater trochanter and caput femori were used as lower and upper reference levels, respectively. To define the ROI in humeri, the distal metaphyseal growth plate and the fusion zone between the greater tuberosity and caput humeri were used. Finally, the most proximal and distal 20% of the ROI were subjected to 3D bone architecture analysis to investigate trabecular structures. The 3D algorithms were used in CT Analyser to calculate the following parameters: trabecular number (Tb.N.), trabecular separation (Tb.Sp), trabecular thickness (Tb.Th) and bone volume fraction (BV/TV). For the subsequent assessment of cortical structures, areas between upper and lower reference levels were divided into 10 sections. The cortical ROI was located in the middle of the diaphysis and contained 10% of the upper and 10% of the lower reference levels, respectively. Afterwards, the diaphyseal area was subjected to a 2D analysing algorithm to measure the parameters cortical thickness (Ct.Th) and cortical area fraction (Ct.Ar/Tt. Ar.) (Bouxsein et al., 2010; Behrendt et al., 2016). In order to eventually determine bone mineral density (BMD) in the diaphysis, attenuation coefficient of the phantoms of known density (0.25 and 0.75 g/cm^3^) were used and the BMD was calculated automatically by algorithms.

### 2.7 Blood sampling and analyses of bone turnover markers

Blood samples were taken by saphenous vein puncture before the start of the training phase for later determination of basal values (Figure 1). For this purpose, the mice were fixed as previously described (PerkinElmer, Massachusetts, United States) (Hem et al., 1998). The thigh was shaved distally to the knee, the veins were tourniquetted by manual compression, punctured by a 25G needle (B. Braun SE, Melsungen Germany) and blood was collected into an EDTA microvette (Sarstedt, Nümbrecht, Germany). Samples were temporarily stored on ice. Subsequently, plasma was separated *via* centrifugation (Eppendorf SE, Germany) and stored at −80°C until further analysis. At the end of each training phase, mice were anesthetized by intraperitoneal injection of ketamine/xylazine (100 mg ketamine and 5 mg xylazine/kg body weight) for final blood sampling. In detail, blood was collected by puncturing the retrobulbar venous plexus, plasma was separated and preserved as described above. Before removal of organs and bones, the animals were euthanized *via* cervical dislocation. To assess bone metabolism, TRACP5b, as a marker of bone resorption and osteoclast activity, and PINP, as a marker of bone formation, were analysed *via* an ELISA for TRAcP 5b (Mouse Trap, Immunodiagnostic Systems Ltd., Boldon, United Kingdom) and an EIA for PINP (Rat/Mouse PINP EIA, Immunodiagnostic Systems Ltd., Boldon, United Kingdom). Both kits were used according to the manufacturer’s instructions. Due to a limited sample volume, the tests were only performed once. Photometric measurements of TRAcP ELISA and PINP EIA were performed using the Infinite M200 automated plate reader running with the iControl software v1.9 (Tecan Trading AG, Switzerland) at an absorbance of 405 nm with reference at 650 nm. Subsequently, the concentrations of our samples were estimated for TRAcp5b using a 3-parameter logistic regression model for calibration. In order to extrapolate concentration for high absorbance values readings of absorbance at 450 were naturally logarithmized (ln) and the resulting linear regression was used for further estimations. Back transformation using e^ln(conc) was used to calculate the concentrations of PINP in ng/mL. In both cases, RStudio 1.2.5033 software (version 3.5.1, RStudio Inc. Boston, MA, United States) and Microsoft Excel (Microsoft, Redmond, WA, United States) were used.

### 2.8 Three-point bending test

After removal, the left femora were soaked in 0.9% NaCl and wrapped, placed in Eppendorf tubes and frozen at −20°C until further analyses. 24 h before the measurements, samples were thawed and continuously kept moist using 0.9% sodium chloride solution to protect them from exsiccation. First, the exact dimensions of the bones were collected using a caliper gauge (Vogel GmbH, KevelaerGermany) for the subsequent calculations. The femora were then placed in the three-point bending machine with the condyles facing downwards so that the femoral mid-shaft was centred with a span of 6 mm on the test punch. The three-point bending machine was equipped with a 500N pressure load cell (zwickiLine Z2.5, Zwick GmbH, Ulm, Germany) and gradually increased the bending force applied to the femur by 1 mm/min during the course of the test. The increase of the bending force was either stopped manually or automatically by the measuring unit when the bone broke. Subsequently, the recorded load-displacement curves could be used to calculate bending strength (MPa), maximum load (N), breaking load (N) and Young`s modulus (MPa), taking into account the bones’ elliptical cross-section (Grote und Feldhusen 2007).

### 2.9 Statistics

The statistical analyses in our experiments were performed using IBM SPSS Statistics 27 software (IBM, NY, United States). The Shapiro-Wilk test was used to examine the normal distribution of the data. Due to small sample sizes we used non-parametric tests only. Comparisons between two groups (OVX vs. non-OVX or trained vs. untrained) were performed using Mann-Whitney U-tests and Kruskal–Wallis with *post hoc* tests for multiple comparisons. Pairwise comparisons (TRAcP concentrations at experimental day 6 vs. at sacrifice) were done *via* Wilcoxon Signed-Rank-test. Values of p lower than 0.05 were considered significant. Finally, the experimental results were presented graphically using SigmaPlot 13.0 (Systat Software, CA, United States).

## 3 Results

### 3.1 Treadmill training led to increased running speeds

Four augmentation run tests (ART) dispersed over the length of the experiments served to adapt the interval training and to evaluate the impact of the training. Individual maximum running velocities (Vmax) before the start of the training period (ART1) ranged between 0.22 and 0.37 m/s and were comparable between all groups (Table 1). Likewise, at any given ART thereafter, mean Vmax between groups were comparable as shown by *p*-values ranging from 0.3691 to 0.9288. However, all groups increased their mean Vmax from ART1 to ART4. While this increase was only marginal for groups IV (20° incline) and VI (interval training), it was significant for groups III (no decline/incline) and V (20° decline). The highest results across all running groups was achieved by group V. In summary, all training regimes led to increased running speeds and did so with comparable efficiency.

**TABLE 1:** Treadmill training led to increased running speeds

group	Vmax-ART 1 mean ± SD [m/s]	Vmax-ART 2 mean ± SD [m/s]	Vmax-ART 3 mean ± SD [m/s]	Vmax-ART 4 mean ± SD [m/s]	*p*-value [H-test]
III: endurance training no incline (n = 8)	0.274 ± 0.04	0.289 ± 0.03	0.315 ± 0.04	0.35 ± 0.03	**0.006**
IV: endurance training 20° incline (n = 9)	0.284 ± 0.05	0.302 ± 0.06	0.322 ± 0.05	0.336 ± 0.05	0.152
V: endurance training 20° decline (n = 6)	0.280 ± 0.05	0.325 ± 0.03	0.308 ± 0.04	0,372 ± 0.03	**0.009**
VI: interval training (n = 6)	0.290 ± 0.05	0.315 ± 0.03	0.322 ± 0.04	0.352 ± 0.04	0.111
*p*-value [H-test]	0.967	0.375	0.665	0.404	

Bold values are statistically significant (*p* < 0.05).

### 3.2 Treadmill training led to significant increases in heart weight

**FIGURE 2:**
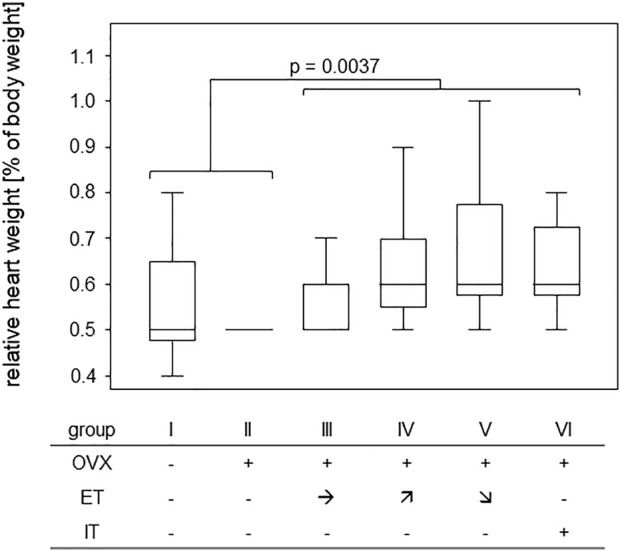
Treadmill training led to significant gains in heart weight. Box plots show the ratios of heart to body weight for the various training groups. Comparison between untrained (groups I and II) and trained groups (groups III-VI) was performed *via* Mann-Whitney U-Test and statistically significant difference is indicated by the *p*-value.

In order to assess any training effect on the cardiovascular system, heart weights were measured post-mortem and were calculated in relation to the respective body weight. While median ratios of heart to body weight were 0.5% for the untrained groups I-II, they were 0.6% for the trained groups III-VI. A Mann-Whitney test comparing trained and untrained animals resulted in a *p*-value of 0.0037 confirming that 14 weeks of treadmill training led to significant gains in heart weight (Figure 2).

### 3.3 Treadmill training significantly increased the muscle volumes of the lower extremities

**FIGURE 3:**
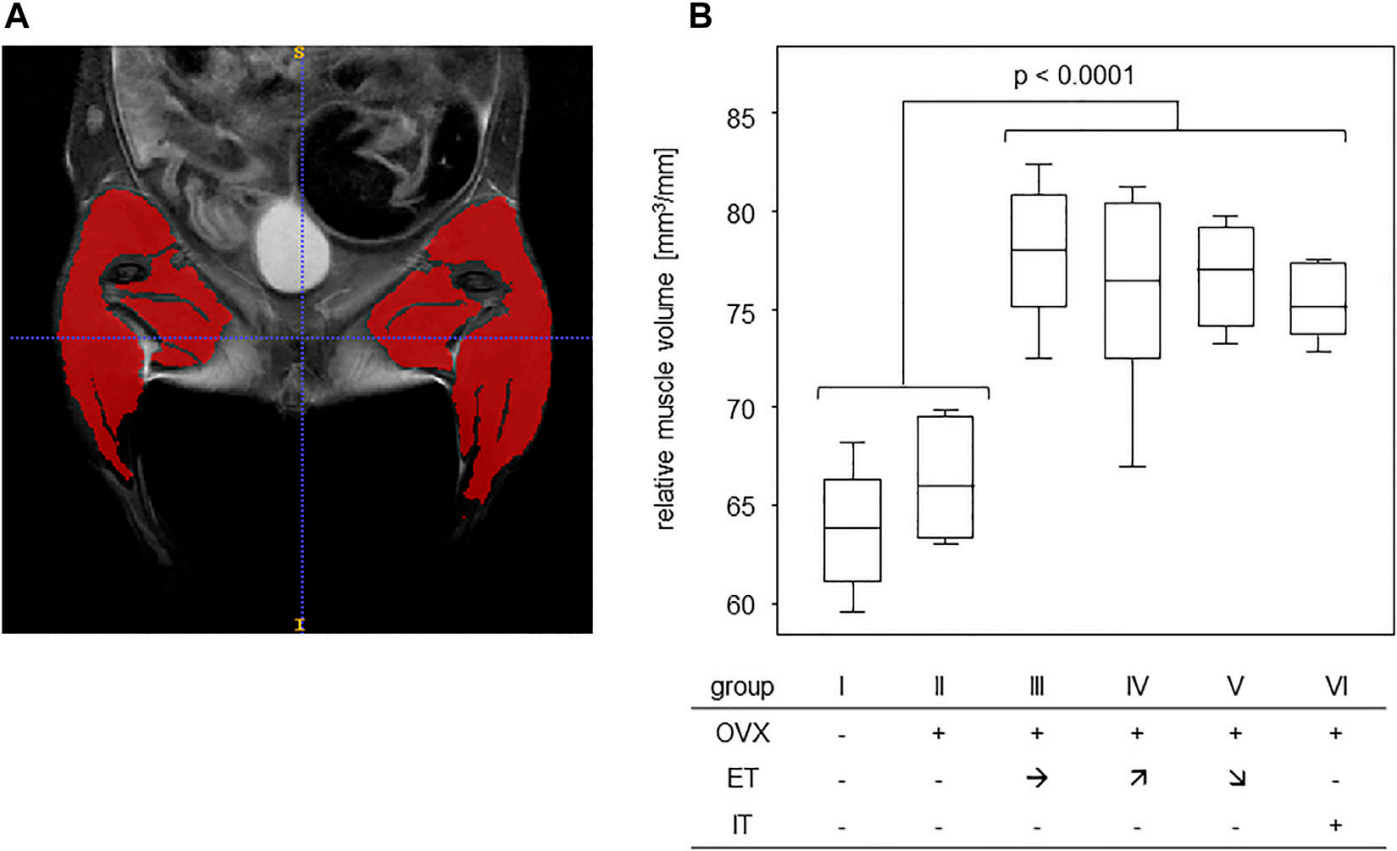
Treadmill training increased muscle volumes of the lower extremities. **(A)** shows an exemplary T2 TurboRARE illustration of an abdomen, pelvis and lower extremities and marks the muscle tissues of the lower extremities by ITK snap. **(B)** presents the muscle volumes in relation to femur lengths of groups that were either untrained (groups I-II) or trained (groups III—VI). Statistics were calculated by Mann-Whitney U-test and statistically significant difference is indicated by the *p*-value.

In order to assess any training effect on the musculature, MRI scans of the lower extremities and pelvic regions were performed (Figure 3A). In detail, the scans served to assess the muscle volumes of the lower extremities which were then—in order to compensate for different sizes - calculated in relation to the respective femur length. The results yielded cross sectional areas and Figure 3B summarizes the results for all experimental groups. Comparing the cross sectional areas between the untrained groups I and II (median of 64.2 mm^2^) and the trained groups III-VI (median of 76.3 mm^2^) resulted in a statistically significant difference (Figure 3B).

### 3.4 Treadmill training did not compensate for OVX-induced loss of bone stability and elasticity

**FIGURE 4:**
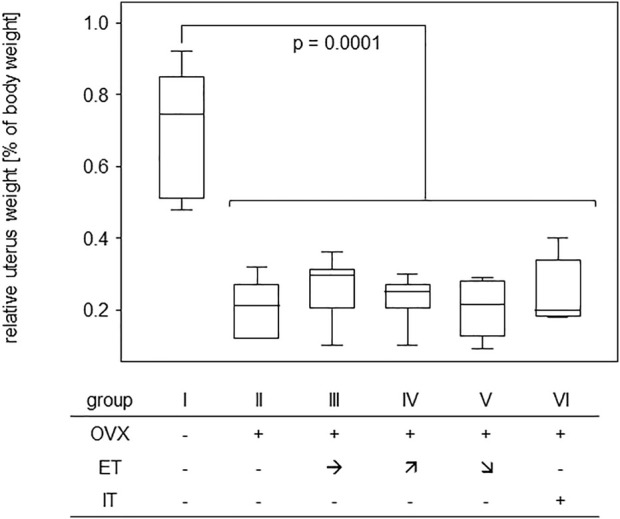
Effective OVX was confirmed by significant weight reduction of uteri. Box plots represent uterus weights compared to body weights for all groups. Statistics were calculated by Mann-Whitney U-test and statistically significant difference is indicated by the *p*-value.

**FIGURE 5:**
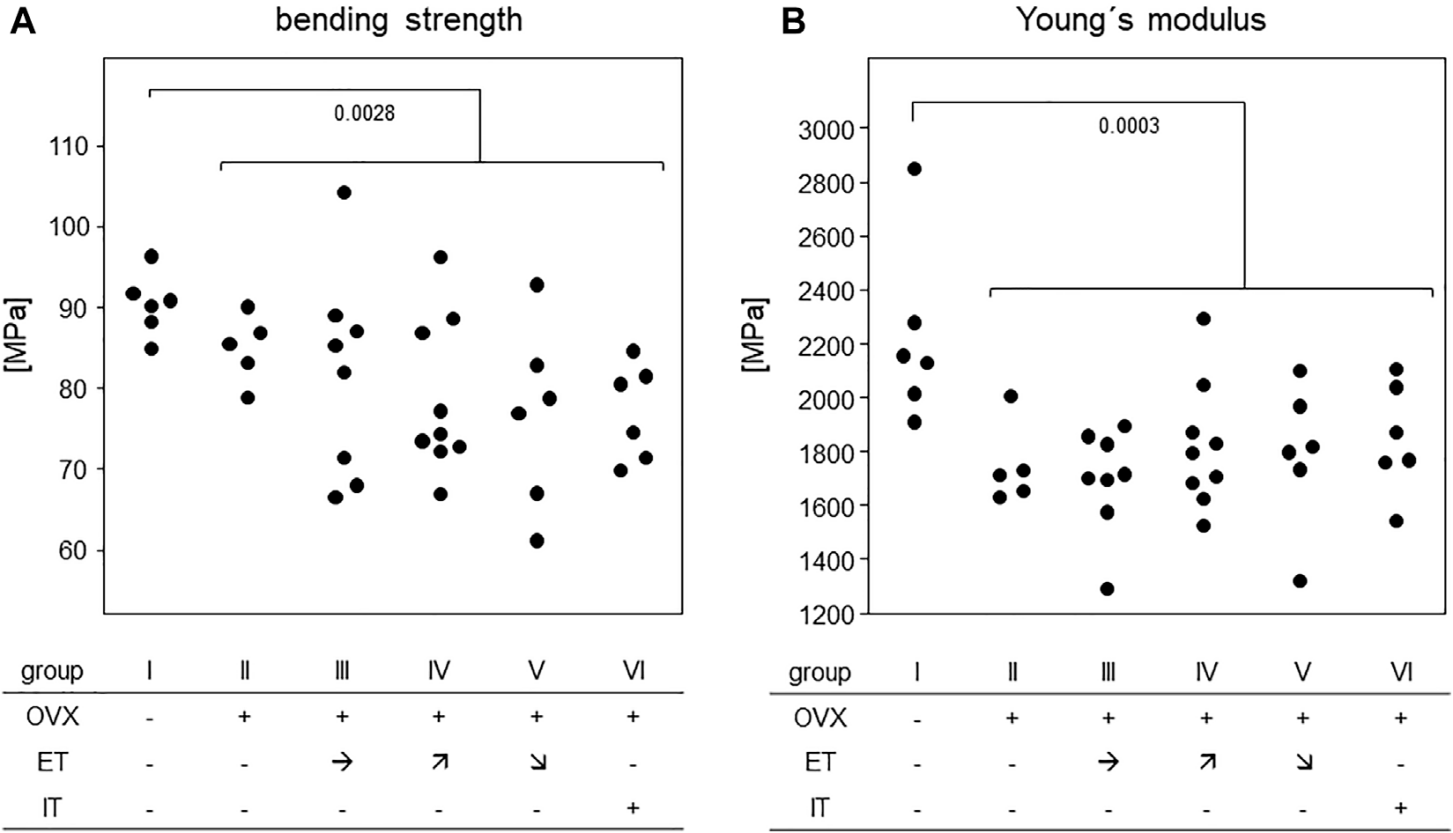
Treadmill training did not compensate for OVX-induced loss of bone stability. Dot blots summarize **(A)** the bending strength and **(B)** Young`s Modulus. Statistics were calculated by Mann-Whitney U-test and statistically significant differences are indicated by *p*-values.

In order to assess the efficiency of OVX, post-mortem uterus weights were taken and compared between all experimental groups. Results are presented in Figure 4 and show a significant difference between the sham operated group and all others confirming successful procedure. For analysis of bone stability, the femora were subjected to three-point bending tests. The results presented as bending strength and Young’s modulus are summarized in Figure 5. While the sham group I (no OVX, no training) exhibited highest means for bending strength and Young’s modulus, respectively, OVX resulted in a significant reduction for both. However, there were no significant differences between any of the trained groups and the non-trained OVX control. In summary, OVX led to a reduction of both, flexural strength and elasticity, and neither of the training regimens managed to prevent loss of bone integrity.

### 3.5 Treadmill training did not prevent OVX-induced bone loss

**FIGURE 6:**
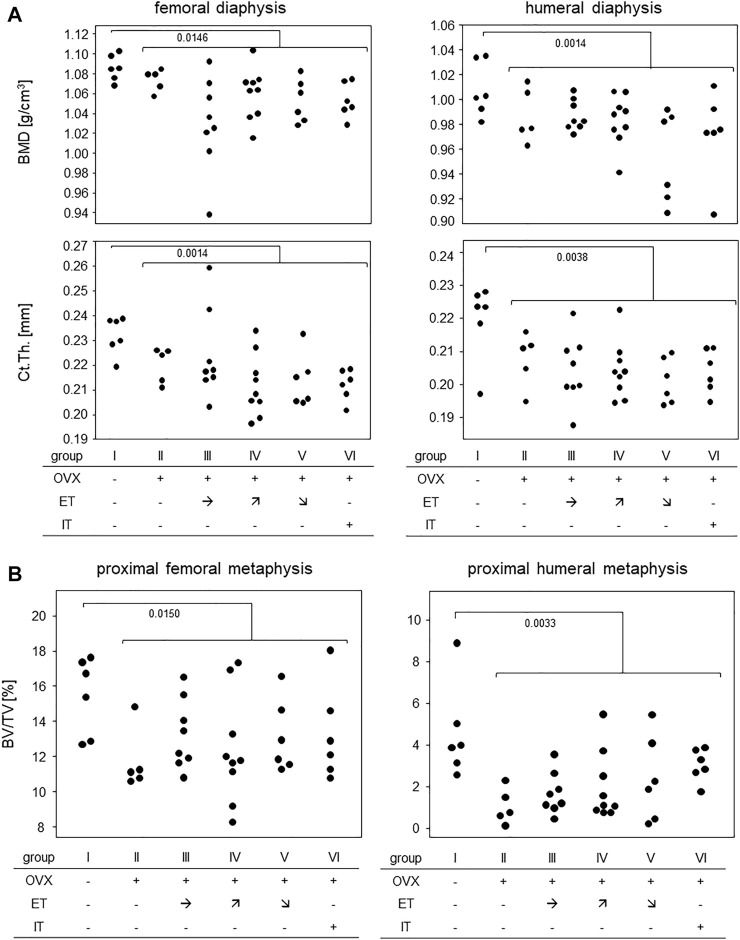
Treadmill training did not prevent OVX-induced bone loss. **(A)** shows bone mineral density and cortical thickness of the femoral (left panels) and humeral (right panels) diaphysis. **(B)** shows BV/TV in the proximal metaphyses of femora (left panel) and humerus (right panel). Statistics were calculated by Mann-Whitney U-test and statistically significant differences are indicated by *p*-values.

Measurements of cortical bone parameters were collected in the diaphyses of both, femora and humeri and the results are summarized in Figure 6A: The highest mean bone mineral density (BMD) for both extremities were found in group I (no OVX, no training). OVX resulted in a significant reduction in BMD, but there was no statistically significant difference between the trained animals (group III-VI) and group II. Likewise, cortical thicknesses of femur and humerus were highest in the SHAM group with median values of 0.23 and 0.22 mm, respectively (Figure 6A). Again, there was no difference between any of the trained groups and group II.

Figure 6B summarizes the results for the trabecular bone parameters assessed in the distal metaphyses of femora and humeri. As with the cortical parameters, highest means for BV/TVs were observed in the SHAM group with medians of 16.0% for femora and 3.9% for humeri, respectively. All OVX groups yielded significantly lower means with none of the trained groups differing from control group II (Figure 6B). Additional measurements obtained by CT are available in the supplements. In summary, OVX led to a loss of cortical and trabecular bone parameters and none of the treadmill training regimen was able to prevent this process.

### 3.6 Treadmill training did not impact on bone metabolism

**FIGURE 7:**
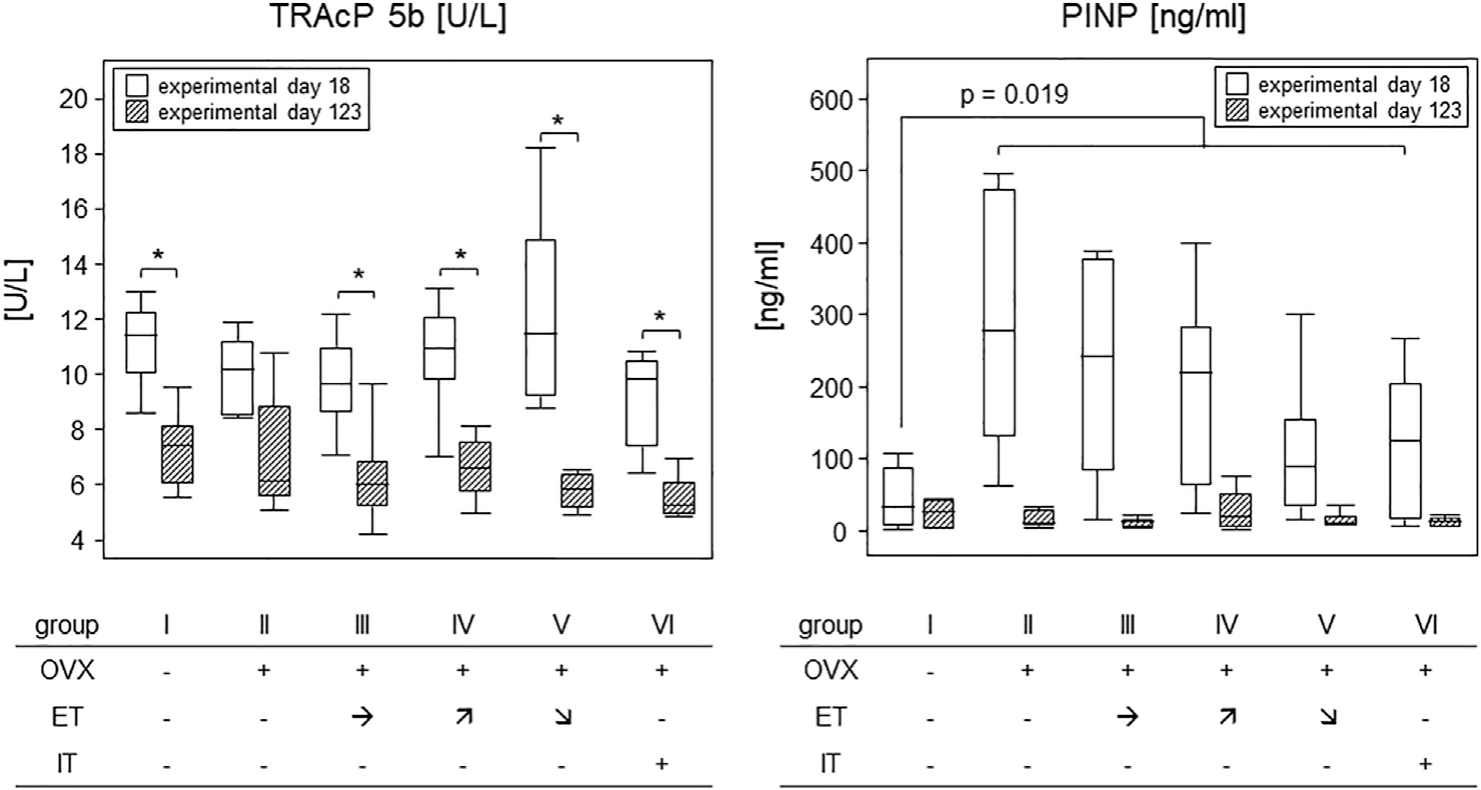
Treadmill training did not impact on bone metabolism. Box plots compare serum TRAcP 5b (left panel) and PINP (right panel) concentrations on experimental day 18 and at sacrifice. To describe changes in TRAcP concentrations (left panel), statistics were calculated by Wilcoxon signed-rank test, asterisks indicate statistically significant differences (*p < 0.05). For changes in PINP concentrations on experimental day 6 (right panel), the sham group I was compared to all OVX groups (II—VI) *via* Mann-Whitney U-test. For details see text.

To investigate how OVX and treadmill training impacted on the metabolic activity of osteoclasts and osteoblasts, serum TRAcP and PINP were analysed. In detail, we compared experimental day 18–after OVX yet before the start of the treadmill training - to the endpoint of the experiments at sacrifice (see Figure 1). Figure 7 summarizes our results. While TRAcP-values were comparable among all experimental groups at both time points, values did differ significantly between the early and late time points (left panel). At the early time point, serum values for TRAcP 5b ranged from means of 9.2 U/L in group VI to 12.2 U/L in group V however, they dropped significantly to means ranging between 5.5 (group V) and 7.3 U/L (group I) at the experimental end point. *p*-values describing these differences pairwise were 0.028 (group I); *p* = 0.08 (group II) 0.017 (group III) *p* = 0.011 (group IV), 0.028 and *p* = 0.046 (group VI). In summary, osteoclast metabolic activity was affected by age, but unaffected by OVX or treadmill training.

The situation was different for PINP. OVX led to an almost 10-fold and significant rise in PINP levels within 18 days following surgery (right panel). As the treadmill training had not begun yet, all OVX mice were calculated as one group and showed a statistically significant increase from a median 1.2 ng/ml in group I to 11.6 ng/ml for all OVX mice. The *p*-value resulting from a Mann-Whitney test was 0.019. At sacrifice, serum PINP concentrations were again comparable among all groups with a median value of 1.7 ng/ml (Figure 7). In summary, osteoblast metabolic activity was transiently elevated by OVX but was unaffected by treadmill training.

## 4 Discussion

We here explored treadmill training for its capacity to prevent OVX induced bone loss. In detail, we compared increased axial loading *via* a 20° decline of the treadmill, to increased tensile forces *via* a 20° incline to endurance training with no gradient to high intensity interval training. We confirmed efficient OVX followed by deterioration of bone microstructures and mechanical properties and we validated our training *via* improved running velocities and increased muscle volumes of both, heart and lower extremities. These validations are in line with previous publications (Kaplan et al., 1994; Gibb et al., 2017; Latza et al., 2020). A distinctive feature of our work is the CT-based analysis of femora and humeri in order to control for our experimental animals being quadrupeds.

In short, the analysis of bone mechanical stability following treadmill training was rather disillusioning. Despite an extended training period of 14 weeks, we here confirmed earlier results with a shorter training period from our own group demonstrating that treadmill training by itself did not suffice to prevent OVX-induced deterioration of bending strength and Young’s modulus (Latza et al., 2020). Considering that bone stability is mainly dependent on cortical parameters (Holzer et al., 2009), these findings are in line with our observation that cortical bone parameters of front and hind legs deteriorated in all OVX mice, again without any benefits from treadmill training. Moreover, our CT analyses confirmed that OVX also led to a loss of trabecular bone which could not be prevented by treadmill training. Negative as these results are, they are in line with a number of previous experiments that aimed to improve bone parameters in young and healthy mice (Koenen et al., 2017; Hollinski et al., 2018) or prevent OVX induced bone loss (Latza et al., 2020). Our research group so far analysed different mouse strains, compared purely aerob to more strenuous training regimen with anaerob peaks, investigated varying training durations and assessed the impacts of axial loading vs. tensile strain by introducing various inclines and declines. However, irrespective of whether training periods lasted 5 or 14 weeks, we never observed any clear cut positive effect on the bone that would immediately be translated into a suggestion for osteoporosis prone patients. Our results suggest that increases in muscle volume of the heart and the extremities are not paralleled by the retention of bone integrity. We are aware though that the literature holds conflicting results as to training effects on the bone and can only speculate about these discrepancies (Iwamoto et al., 1998; Wu et al., 2001). Among the arguments at hand are different genetic backgrounds, diverse experimental set-ups and varying read-outs (Wallace et al., 2015; Zhang et al., 2020).

Comparable to structural and mechanical bone characteristics, metabolic bone parameters were also unaffected by any of the training programs. Nevertheless both, PINP as a marker of bone formation and TRAcP-5b as a marker for bone resorbing osteoclast activity, showed altered expressions over the course of the experimental phase. In detail, PINP was transiently increased after OVX and again down to baseline at the end of the experiments, 14 weeks later. Likewise, Rissanen et al. previously described that PINP in rats significantly increased during the first 2 weeks after OVX and returned to sham level at 8 weeks (Rissanen et al., 2008). Collectively, these findings foster the expectation of a transient increase in bone mass rather than a decrease. However, Rissanen et al. also described an OVX induced and permanent increase in carboxy-terminal collagen crosslinks (CTX) that reflects ongoing bone resorption and holds the potential for disturbing the bone remodeling balance towards resportion. In contrast, our TRAcP levels were unaffected by OVX and even decreased over the course of the experiments. These findings are conflicting as transiently elevated PINP and decreasing TRAcP-levels cannot be reconciled with a net loss of bone mass, deterioration of bone integrity and osteoporosis. The possibility remains though, that serum measurements only inaccurately reflect processes at the bone surface and that metabolic requirements for homeostasis in the light of an osteoporotic bone mass are difficult to calculate.

In summary, our results challenge the idea that treadmill training by itself is suitable to stop OVX-related bone loss. For instance, combining endurance and athletic exercises may turn out more beneficial for the bone. Indeed, the HiRiT program of an Australian study included deadlifts, overhead presses, squats and pull-ups with drop landings and led to a significant improvement of the bone structure (Watson et al., 2018). Even though encouraging, these experiments are difficult to model in animal experiments. Alternatively, the diet could be supplemented with extra calcium and vitamin D in order to investigate if the combination with treadmill training will favour bone accrual. Finally, as an additional preventive measure, treadmill training may allow for a lower minimum therapeutic dose and an associated reduced risk of adverse drug reactions, in addition to the overall improvement in cardiovascular risk profile.

## Data Availability

The original contributions presented in the study are included in the article/Supplementary Material, further inquiries can be directed to the corresponding author.
