# Supplementary material for: Prolonged treadmill training is not able to prevent ovariectomy-induced bone loss
Source: Front Physiol. 2022 Dec 16;13:1078857. doi: 10.3389/fphys.2022.1078857 (PMC9800899; doi:10.3389/fphys.2022.1078857)
Supplement: Supplementary file 1 [file DataSheet1.PDF]

Table 1: Supplementary µCT-data of femur

group	SMI Met. prox. mean ± SD	Conn. Met. prox. mean ± SD	Conn.D. Met. prox. mean ± SD [1/mm^3]	SMI Met. dis. mean ± SD	Conn. Met. dis. mean ± SD	Conn.D. Met. dist. mean ± SD [1/mm^3]	Tb.th. Met. prox. mean ± SD [mm]	Tb.N. Met. prox mean ± SD [1/mm]	Tb.sp. Met. prox. mean ± SD [mm]	Tb.th. Met. dis. mean ± SD [mm]	Tb.N. Met. dis. mean ± SD [1/mm]	Tb.sp. Met. dis. mean ± SD [mm]
I:	1.91 ± 0.13	92.33 ± 18.95	49.77 ± 9.2	3.014 ± 0.157	17.17 ± 8.61	8.239 ± 4.22	0.101 ± 0.005	1.517 ± 0.157	0.477 ± 0.053	0.0650 ± 0.009	0.277 ± 0.081	0.513 ± 0.155
II:	2.01 ± 0.14	74.0 ± 17.76	36.80 ± 10.12	2.540 ± 0.202	18.80 ± 8.16	9.488 ± 3.89	0.094 ± 0.004	1.251 ± 0.229	0.472 ± 0.62	0.0659 ± 0.007	0.365 ± 0.084	0.676 ± 0.172
III:	2.04 ± 0.14	83.5 ± 19.59	45.00 ± 10.34	2,520 ± 0.238	10.75 ± 10.31	5.320 ± 5.76	0.097 ± 0.004	1.363 ± 0.213	0.483 ± 0.066	0.0628 ± 0.006	0.265 ± 0.205	0.753 ± 0.182
IV:	1.97 ± 0.21	66.22 ± 23.74	37.93 ± 12.83	2.611 ± 0.276	11.89 ± 9.68	6.020 ± 5.50	0.092 ± 0.003	1.358 ± 0. 249	0.441 ± 0.047	0.0635 ± 0.003	0.254 ± 0.189	0.733 ± 0.179
V:	1.87 ± 0.16	89.67 ± 11.9	43.35 ± 6.97	2.556 ± 0.256	16.33 ± 9.79	7.316 ± 5.59	0.096 ± 0.007	1.375 ± 0.204	0.510 ± 0.063	0.0604 ± 0.005	0.294 ± 0.198	0.788 ± 0.180
VI:	1.84 ± 0.18	77.33 ± 13.37	41.25 ± 7.06	2.547 ± 0.227	10.50 ± 9.98	5.491 ± 5.75	0.091 ± 0.009	1.459 ± 0.138	0.451 ± 0.70	0.0635 ± 0.005	0.263 ± 0.197	0.732 ± 0.177
p-value [H-test]	0.36	0.256	0.36	**0.015**	0.505	0.656	0.151	0.449	0.582	0.733	0.795	0.135

Table 2: Supplementary µCT-data of humerus

group	SMI Met. prox. mean ± SD	Conn. Met. prox. mean ± SD	Conn.D. Met. prox. mean ± SD [1/mm^3]	SMI Met. dis. mean ± SD	Conn. Met. dis. mean ± SD	Conn.D. Met. dist. mean ± SD [1/mm^3]	Tb.th. Met. prox. mean ± SD [mm]	Tb.N. Met. prox mean ± SD [1/mm]	Tb.sp. Met. prox. mean ± SD [mm]	Tb.th. Met. dis. mean ± SD [mm]	Tb.N. Met. dis. mean ± SD [1/mm]	Tb.sp. Met. dis. mean ± SD [mm]
I:	2.589 ± 0.154	17.50 ± 11.87	18.75 ± 12.1	2.446 ± 0.17	18.17 ±5.90	70.17 ± 20.99	0.067 ± 0.005	0.672 ± 0.263	0.486 ± 0.126	0.09 ± 0.013	0.76 ± 0.144	0.30 ± 0.022
II:	2.624 ± 0.178	5.80 ± 10.98	6.38 ± 11.4	2.374 ± 0.15	15.00 ± 6.79	43.13 ± 25.80	0.054 ± 0.006	0.182 ± 0.278	0.729 ± 0.148	0.10 ± 0.011	0.79 ± 0.147	0.32 ± 0.022
III:	2.630 ± 0.220	7.00 ± 11.43	8.26 ± 11.77	2.329 ± 0.16	11.63 ± 5.61	33.70 ± 22.18	0.058 ± 0.010	0.271 ± 0.365	0.655 ± 0.165	0.08 ± 0.011	0.82 ± 0.082	0.33 ± 0.019
IV:	2.650 ± 0.217	9.44 ± 4.51	10.77 ± 4.92	2.409 ± 0.18	14.22 ± 5.11	43.69 ± 21.19	0.065 ± 0.009	0.283 ± 0.229	0.664 ± 0.145	0.10 ± 0.011	0.79 ± 0.096	0.33 ± 0.021
V:	2.703 ± 0.217	11,00 ± 3.89	11.91 ± 3,91	2.354 ± 0.19	17.33 ± 4.64	55.72 ± 20.05	0.056 ± 0.008	0.395 ± 0.202	0.605 ± 0.107	0.09 ± 0.011	0.99 ± 0.100	0.30 ± 0.017
VI:	2.638 ± 0.219	11,00 ± 3.79	12.19 ± 3,75	2.344 ± 0.21	16.17 ± 4,40	46.58 ± 20.23	0.069 ± 0.008	0.440 ± 0.147	0.634 ± 0.053	0.09 ± 0.011	0.90 ± 0.102	0.32 ± 0.017
p-value [H-test]	0.982	0.348	0.464	0.945	0.637	0.092	0.05	**0,012**	**0.035**	0.372	0.264	0.293
